# Disruptive DNA Intercalation Is the Mode of Interaction Behind Niacinamide Antimicrobial Activity

**DOI:** 10.3390/microorganisms13071636

**Published:** 2025-07-10

**Authors:** Michal Rasis, Noa Ziklo, Paul Salama

**Affiliations:** Innovation Department, Sharon Personal Care Ltd., Eli Horovitz St. 4, Rehovot 7608810, Israel; michalr@sharonpc.com (M.R.); noa.ziklo@sharonpc.com (N.Z.)

**Keywords:** niacinamide, DNA interaction, intercalation, antimicrobial

## Abstract

Niacinamide was recently shown to directly interact with bacterial DNA and interfere with cell replication; niacinamide mode of interaction and efficacy as a natural anti-microbial molecule were also described. The aim of this study is to elucidate the exact binding mechanism of niacinamide to microbial DNA. Intercalation is a binding mode where a small planar molecule, such as niacinamide, is inserted between base pairs, causing structural changes in the DNA. Melting curve analysis with various intercalating dyes demonstrated that niacinamide interaction with bacterial DNA reduces its melting temperature in a linear dose-dependent manner. Niacinamide’s effect on the melting temperature was found to be % GC-dependent, while purine stretches were also found to influence the binding kinetics. Finally, fluorescent intercalator displacement (FID) assays demonstrated that niacinamide strongly reduces SYBR Safe signal in a dose-dependent manner. Interestingly, competition assays with a minor groove binder also reduced Hoechst signal but in a non-linear manner, which can be attributed to strand lengthening and unwinding following niacinamide intercalation. Taken altogether; our results suggest a “disruptive intercalation” as the mode of interaction of niacinamide with bacterial DNA. Formation of locally destabilized DNA portions by niacinamide might interfere with protein–DNA interaction and potentially affect several crucial bacterial cellular processes, e.g., DNA repair and replication, subsequently leading to cell death.

## 1. Introduction

Niacinamide is a water-soluble amide form of niacin, with both compounds representing two forms of vitamin B3. Naturally occurring in both plant- and animal-derived food products, niacinamide is also extensively utilized in cosmetic skincare formulations due to its multiple beneficial properties. Recently, Ziklo et al. demonstrated for the first time that niacinamide directly interacts with bacterial DNA, causing interference with DNA replication and cell cycle arrest [1]. Direct antimicrobial mechanisms of action were previously described for niacinamide in several studies, both in eukaryotes and prokaryotes, mainly via the inhibition of the NAD^+^-dependent deacetylase–protein family, called sirtuins [2,3,4]. However, historically, niacinamide’s antimicrobial activity has been primarily attributed to its ability to stimulate the host immune response. This includes the activation of neutrophils and the induction of antimicrobial peptide (AMP) synthesis, mechanisms that provide long-lasting protection against bacterial infections [5,6].

Small, non-covalent DNA-binding molecules can be largely categorized as intercalators and groove binders [7]. The driving binding interactions can include electrostatic, hydrophobic, allosteric, hydrogen bonding, and/or van der Waals interactions [8]. Intercalating agents, which bind DNA by inserting aromatic moieties between adjacent DNA base pairs, are characterized as heterocyclic ring molecules that resemble the ring structure of base pairs and are commonly used as DNA stains (e.g., ethidium bromide (EtBr), acridine orange (AO)) [9]. Many minor groove binders are typically crescent-shaped, allowing them to fit into the concavity of the minor groove; classic examples include distamycin and Hoechst dye [7]. Unlike minor groove binders, major groove binders are structurally diverse, often in the form of large planar molecules or have bulky side groups designed to occupy the more spacious major groove [10]. Minor grooves DNA binding involving specific sequences, mostly AT, takes place by means of a combination of directed hydrogen bonding to base pair edges, van der Waals interactions with the minor groove walls, and generalized electrostatic interactions [11,12]. On the contrary, intercalation is usually independent of the DNA sequence context, although a slight GC specificity has been observed [13,14].

DNA intercalators, which can be cationic or neutral, disrupt the continuity of the encoded genome, in contrast to ligands that bind DNA electrostatically or into the major or minor grooves. A DNA double helix can be distorted by intercalating agents, thereby interfering with DNA replication, transcription, and repair [9].

Niacinamide is a small, hydrophilic, planar, aromatic molecule, which is uncharged at physiological pH. It can potentially participate in hydrogen bonding, van der Waals, and π–π interactions. Considering its structural features and biochemical properties, niacinamide could likely be a suitable candidate for DNA intercalation.

Understanding how small molecules interact with DNA has become an active research area at the interface between chemistry and molecular biology [15,16]. Several instrumental techniques were used to decipher such interactions, including UV–Visible spectroscopy and fluorescence-based methodologies [13]. The aim of this study was to identify the mode of interaction of niacinamide with bacterial DNA, which is crucial to our understanding of niacinamide activity as a natural anti-bacterial molecule. Indeed, this qualitative and quantitative understanding is pivotal to us, as our quest for natural antimicrobial substances requires a precise comprehension of the key elements governing their action.

## 2. Materials and Methods

### 2.1. Bacterial Strains

*Pseudomonas aeruginosa* (ATCC 9027) and *Staphylococcus aureus* (ATCC 6538) were obtained from ATCC and cultured according to the manufacturer’s instructions. *Bacillus safensis* (internal laboratory strain collection) was maintained in tryptic soy agar (TSA) (HIMEDIA, Rehovot, Israel).

### 2.2. Chemicals

**Figure 1:**
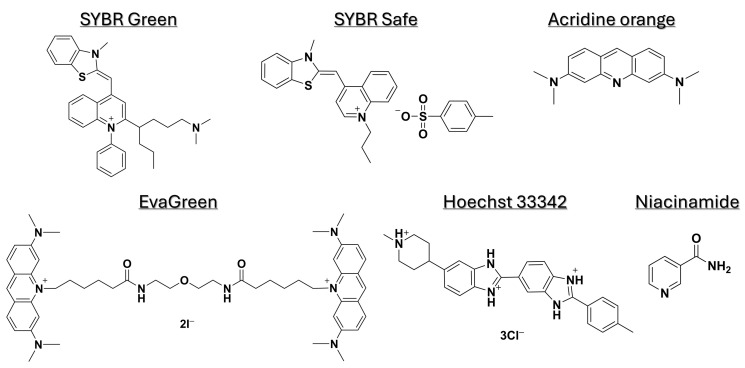
Chemical structures of niacinamide and fluorescent dyes used in this study.

The fluorescent DNA-binding dyes used in this study were SYBR Safe (504.66 g·mol^−1^) (Thermo Fisher Scientific, Lod, Israel), acridine orange (369.96 g·mol^−1^) (Sigma-Aldrich, Rehovot, Israel), EvaGreen (18.01 g·mol^−1^) (Biotium, Fremont, CA, USA), and Hoechst 33342 (561.93 g·mol^−1^) (Abacm, Cambridge, UK). Niacinamide (122.12 g·mol^−1^) was obtained from Tianjin Zhongrui Pharmaceutical, Tianjin, China). The chemical structures of the dyes and niacinamide are shown in Figure 1.

### 2.3. Amplification and Purification of Bacterial Genomic Fragments

Amplification of genomic fragments was performed using Bio-Rad CFX96 real-time polymerase chain reaction (PCR) system (Bio-Rad Laboratories, Haifa, Israel). Shorter bacterial genomic fragments of ≤300 bp were amplified using a standard two-step amplification protocol with SYBR Green master mix (Bio-Rad Laboratories, Haifa, Israel), according to the manufacture’s guidelines. PCR cycling conditions were as follows: initial denaturation at 98 °C for 3 min; 30–35 cycles of 98 °C for 10 s (denaturation), and 60 °C for 15–20 s (annealing, extension, and signal acquisition); followed by a default melting curve analysis step. For larger genomic fragments of >300, a three-step amplification protocol was performed using Taq polymerase PCR master mix (Thermo Fisher Scientific, Lod, Israel), according to the manufacturer’s guidelines. The PCR cycling conditions were as follows: 95 °C for 3 min; 35 cycles of 95 °C for 30 s, 55 °C for 30 s, and 72 °C for 60 s; and a final extension at 72 °C for 5 min. The resulting PCR products were subsequently analyzed using agarose gel electrophoresis. All primers used in this study are listed in Table 1.

Post-amplification, all PCR products were purified using GeneJET PCR purification kit (Thermo Fisher Scientific, Lod, Israel), according to the manufacture’s guidelines. The concentration and purity of the resulting double-stranded DNA (dsDNA) fragments were measured using NanoDrop Lite Plus spectrophotometer (Thermo Fisher Scientific, Lod, Israel). Finally, 50 ng of the purified PCR product were visualized on 2% agarose gel to verify the DNA size and integrity.

### 2.4. Melting Curve Analysis with Niacinamide

All melting curve analysis experiments were performed using a 0.5 °C temperature increment, starting from 65 °C up to 95 °C, with a 5 sec hold time in-between steps. Briefly, the DNA concentration used was 2.3 ng/µL in Tris-EDTA (TE) pH = 7.4 buffer (Thermo Fisher Scientific, Lod, Israel). Each reaction was supplemented with either mono- or bis-intercalating fluorescent dyes to a final concentration of 1X units of SYBR Safe, 9.5 µM acridine orange, and 1X EvaGreen. Then, each reaction was supplemented with various concentrations of niacinamide diluted in TE buffer to final concentrations of 0–6%. For SYBR Green (509.73 g·mol^−1^) melting curve analysis, niacinamide was added directly to the PCR mixture, post-amplification.

### 2.5. Fluorescent Intercalator Displacement (FID) Assays

Fluorescent intercalator displacement (FID) assays were performed using Infinite 200 microplate reader (Tecan, Männedorf, Switzerland), and black 96-well plates (Greiner Bio-One, Kremsmünster, Austria). For each reaction, the DNA concentration used was 0.4 ng/µL in Tris-EDTA (TE) pH = 7.4. Each reaction was supplemented with a SYBR Safe DNA stain to a final concentration of 1.5× units. A fluorescent read (Ex. 502 nm, Em. 530 nm) was performed every 5 min for 30 min until the signal was stabilized. Then, the plate was taken out of the microplate reader, and each well was supplemented with various niacinamide concentrations ranging from 0% to 10%. Following niacinamide addition, a similar kinetic protocol was applied for an additional 1.5 h. DNA diluted in TE and DNA supplemented with niacinamide, without the stain, were used as background control wells. For data analysis, the fluorescent signal of each well was plotted over time, following subtraction of the corresponding background level. The average decrease rate in the fluorescent intercalator signal 30 min following niacinamide addition (i.e., kinetic window) was also calculated and plotted. The results are presented based on three independent experiments.

### 2.6. Niacinamide Competition Assays with a Minor-Groove Binder

Competition assays with a minor groove binder were performed as described in Section 2.5, with several modifications. The dsDNA was supplemented with Hoechst 33342 staining dye to a final concentration of 500 nM, and a corresponding fluorescent read of Ex. 350 nm and Em. 431 nm was performed.

## 3. Results

### 3.1. Niacinamide Interaction with Bacterial DNA Reduces Its Melting Temperature (Tm) in a Dose-Dependent and Linear Manner

**Figure 2:**
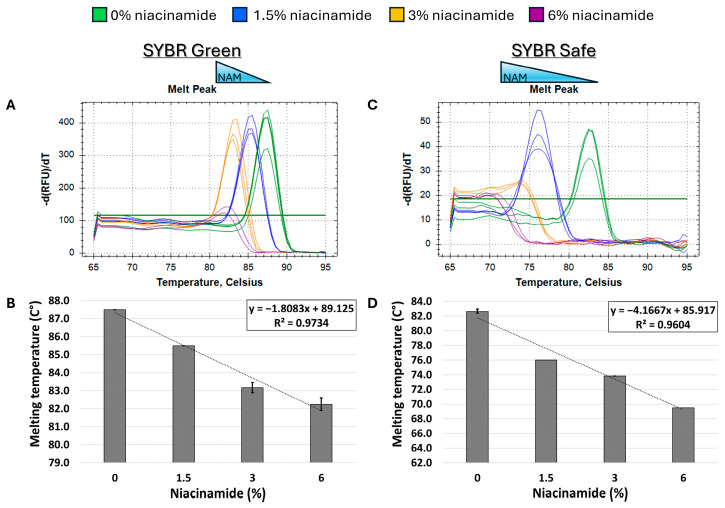
Niacinamide interaction with bacterial DNA reduces its melting temperature (Tm) in a dose-dependent and linear manner. Melting curve analysis of *P. aeruginosa* 102 bp fragment (62% GC) with mono-intercalating fluorescent dyes SYBR Green (**A**,**B**) and SYBR Safe (**C**,**D**). Melting peaks of a representative experiment (**upper panels**) and Tm regression plots (**lower panels**) are presented. The ±SD is based on triplicate wells, with each experiment performed three times.

**Figure 3:**
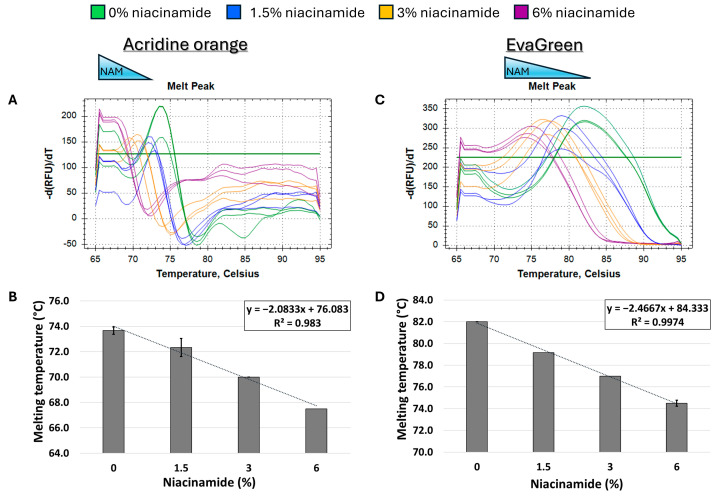
Niacinamide interaction with bacterial DNA reduces its melting temperature (Tm) in a dose-dependent and linear manner. Melting curve analysis of *P. aeruginosa* 102 bp fragment (62% GC) with mono-intercalating fluorescent dye acridine orange (**A**,**B**) and bis-intercalating fluorescent dye EvaGreen (**C**,**D**). Melting peaks of a representative experiment (**upper panels**) and Tm regression plots (**lower panels**) are presented. The ±SD is based on triplicate wells, with each experiment performed three times.

In order to determine the effect of niacinamide interaction on the stability of bacterial dsDNA, a 102 bp fragment of *P. aeruginosa ubi*B (62% GC) was subjected to melting curve analysis using four well-established mono- and bis-intercalating fluorescent DNA stains (Figure 1). *P. aeruginosa* was initially chosen for this analysis based on the relatively low concentration of niacinamide required to inhibit its growth compared to other bacteria [1]. Of note, the Tm of a particular dsDNA fragment is determined by its length, GC content, and the ionic strength of the buffer used. Hence, the Tm of this 102 bp fragment was the highest using SYBR Green compared to the other stains, due to the presence of Mg^++^ ions in the PCR buffer, which stabilize the DNA double helix. Melting curve analysis was performed by incubating equal quantities of dsDNA (50 ng per reaction) with a predetermined stain concentration, based on a preliminary examination of background fluorescence signal elicited by minor interaction with niacinamide and the DNA stain (Supplementary Figure S1). As seen in Figure 2 and Figure 3, the interaction of niacinamide with the DNA fragment resulted in a dose-dependent and linear decrease in the fragment melting temperature (Tm), regardless of the intercalating dye utilized as a reporter. This data strongly indicates that niacinamide has a destabilizing effect on *P. aeruginosa* DNA, as evident by the lower temperature required for DNA denaturation in the presence of niacinamide compared to the control. Moreover, qualitative assessment of the melt peaks suggested that the highest concentrations of niacinamide used may not only result in a shift in the Tm, but also in a decrease in the fluorescent signal (Figure 2). The latter observation further suggests the formation of locally destabilized DNA portions following niacinamide interaction and subsequent dye dislocation, leading to a decrease in Tm and fluorescent signal, depending on niacinamide concentration.

### 3.2. Niacinamide Interaction with Bacterial DNA Is Not Dependent on Fragment Length, but on GC Content

**Figure 4:**
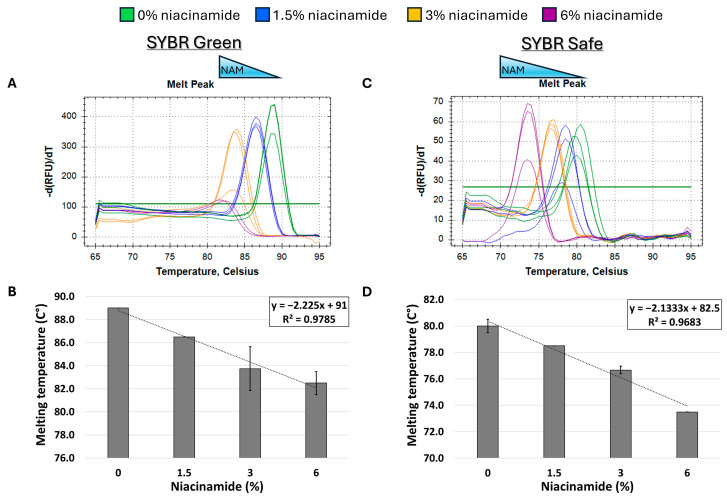
Melting curve analysis of *P. aeruginosa* 198 bp fragment (59% GC) with mono-intercalating fluorescent dyes SYBR Green (**A**,**B**) and SYBR Safe (**C**,**D**). Melting peaks of a representative experiment (**upper panels**) and Tm regression plots (**lower panels**) are presented. The ±SD is based on triplicate wells, with each experiment performed three times.

Given the observed destabilizing effect of niacinamide on Gram-negative *P. aeruginosa* DNA, we set up to test whether this phenomenon is target-specific or more generalized. For that purpose, we performed the same melting curve analysis on an additional *P. aeruginosa* target, using a longer DNA fragment with a similar GC content (~60%). As expected, niacinamide interaction also reduced the Tm of a 196 bp *P. aeruginosa rpo*S fragment in a dose-dependent and linear manner. Comparable results were obtained using SYBR Safe and SYBR Green intercalating dyes (Figure 4B,D), which differ in chemical structures (Figure 1).

**Figure 5:**
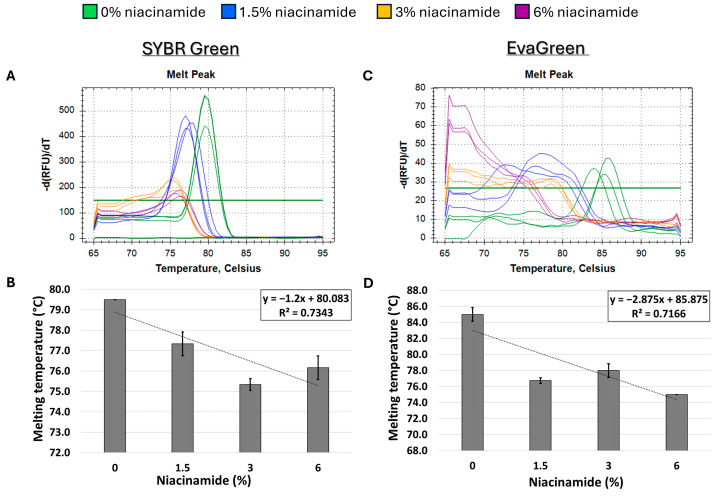
Niacinamide’s interaction with a low-GC bacterial fragment is not linear. Melting curve analysis of *S. aureus* 180 bp *nuc* fragment (34% GC) with mono-intercalating dye SYBR Green (**A**,**B**) and bis-intercalating dye EvaGreen (**C**,**D**). Melting peaks of a representative experiment (**upper panel**) and Tm regression plots (**lower panel**) are presented. Standard deviation is based on triplicate wells, with each experiment performed three times.

**Figure 6:**
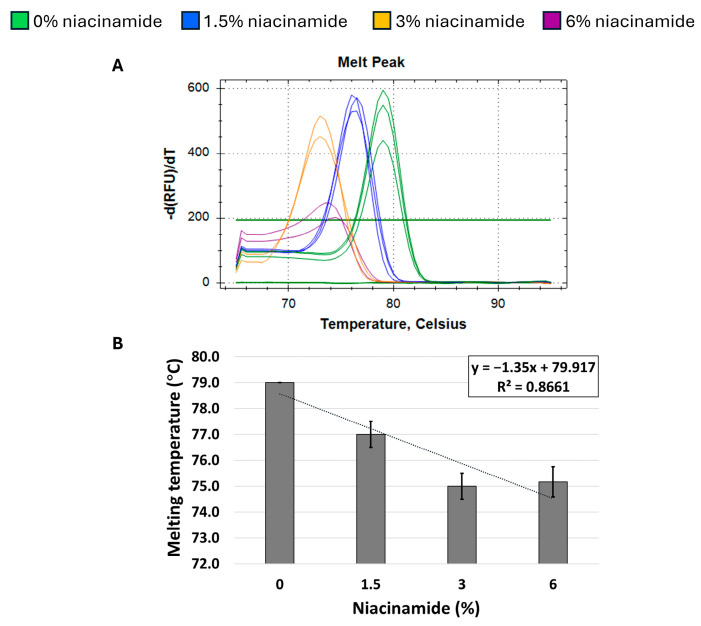
Niacinamide’s interaction with a low-GC bacterial fragment is not linear. Melting curve analysis of *S. aureus* 231 bp *fem*B fragment (31% GC) with SYBR Green. Melting peaks of a representative experiment (**A**) and Tm regression plot (**B**) are presented. Standard deviation is based on triplicate wells, with each experiment performed three times.

Next, we proceeded to explore whether niacinamide’s effect on the Tm of *P. aeruginosa* DNA is predominantly related to its high GC content. Hence, a melting curve analysis of a 180 bp *S. aureus nuc* fragment, with a low GC content (34%) was performed to explore whether niacinamide has a destabilizing effect on the DNA of low-GC fragments as well. As seen in Figure 5, melting curve analysis on the above-mentioned *S. aureus* fragment using both mono-intercalating dye (SYBR Green) and bis-intercalating dye (EvaGreen) resulted in an overall decrease in Tm in the presence of niacinamide. However, this observation was overall dose-dependent, but not linear as was previously demonstrated for high-GC *P. aeruginosa* fragments (Figure 2, Figure 3 and Figure 4). To further verify this result, a second *S. aureus* fragment (*fem*B) with similar GC content (31%) was analyzed, with an overall comparable dose-dependent effect of niacinamide on Tm (Figure 6); however, the effect of niacinamide on the Tm of the *fem*B was more linear (R^2^ = 0.86 vs. R^2^ = 0.71) and the slope of the regression line was higher (−1.35 vs. −1.2), indicating a higher correlation between niacinamide concentration and the Tm of the *fem*B fragment compared to the *nuc* fragment. Taken together, the results presented thus far suggest that niacinamide interaction with bacterial DNA is not sequence length-dependent or target-specific, but GC content-dependent, with a preferable interaction with high-GC-content targets.

### 3.3. Niacinamide Fluorescent Intercalator Displacement (FID) Assays 

**Figure 7:**
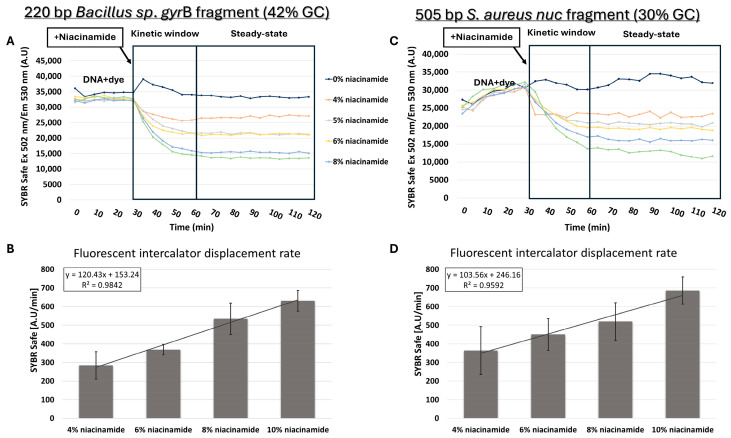
Niacinamide fluorescent intercalator displacement (FID) assays**.** SYBR Safe displacement assay (**upper plots**) with an average intercalator displacement rate 0–30 min post-niacinamide addition (**lower plots**) using 220 bp *Bacillus* fragment (42% GC) (**A**,**B**) and a 505 bp *S. aureus nuc* fragment (30% GC) (**C**,**D**). Plots of a representative FID experiment are shown (**upper panels**). Standard deviations represent three independent experiments.

Based on melting curve analysis, “destabilizing intercalation” and dye dislocation by niacinamide was suggested as a feasible mode of interaction with bacterial DNA. Direct competition with a known intercalator was previously used to further support intercalation as the mode of binding of small ligands to DNA [16]. For that purpose, competition assays were performed between niacinamide and dsDNA bound to the fluorescent intercalator SYBR Safe. Prior to the addition of niacinamide, DNA was incubated for 30 min with a predetermined concentration of the fluorescent intercalator, which was first verified not to have non-specific background fluorescent signal (Supplementary Figure S2). FID assay using 220 bp *Bacillus *sp. *gyr*B fragment (42% GC) clearly demonstrated a dose-dependent decrease in fluorescent signal following the addition of niacinamide, indicating that niacinamide can displace the bound fluorescent intercalator from the DNA (Figure 7A). Furthermore, the average intercalator displacement rate increased linearly with niacinamide concentration (Figure 7B). Surprisingly, similar results, including the linearity of the displacement rate increase, were also obtained for 505 bp *S. aureus nuc* fragment with a low GC content of 30% (Figure 7C,D).

### 3.4. Niacinamide Competition Assay Using Hoechst Minor Groove Binder

**Figure 8:**
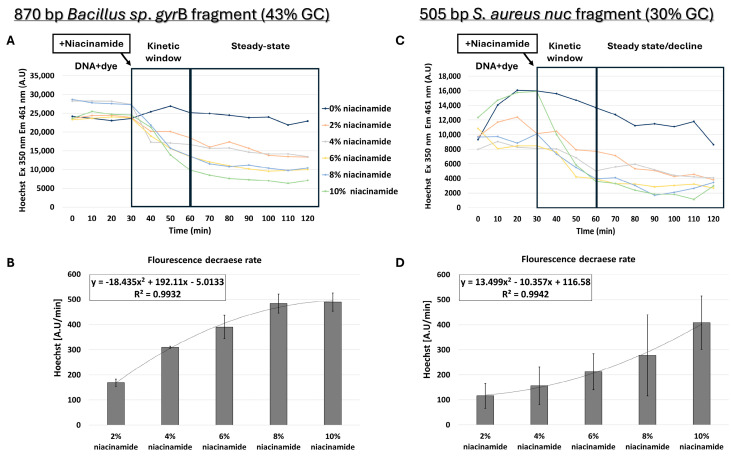
Niacinamide competition assay using Hoechst minor groove binder. Hoechst competition assay with average intercalator displacement rate of 0–30 min post-niacinamide addition using (**A**,**B**) 870 bp *Bacillus* sp. fragment (43% GC) and (**C**,**D**) 505 bp *S. aureus nuc* fragment (30% GC). Plots of a representative competition experiment (**upper panels**) are shown. Standard deviations represent three independent experiments.

Although the structure and biochemical properties of niacinamide, as well as the data presented thus far, support intercalation as the leading mode of interaction of niacinamide with DNA, we also proceeded with testing the possibility of minor groove binding by niacinamide. Although Hoechst dye binds to the minor groove of dsDNA with a preference for AT-rich regions [12], it can also bind to all nucleic acids. To perform competition assays, prior to addition of niacinamide, dsDNA was first incubated for 30 min with a predetermined Hoechst concentration that did not result in a fluorescence signal due to non-specific interaction with niacinamide (Supplementary Figure S2). For minor-groove competition assays, longer fragments of 505 and 870 bp were chosen, as the minor groove covers 5–6 bases per helix turn. Interestingly, the competition assay using 870 bp *Bacillus *sp. *gyr*B fragment (43% GC) also demonstrated a dose-dependent decrease in fluorescent signal following the addition of niacinamide (Figure 8A,B). However, the regression formula showing the relationship between the average fluorescence decrease rate and niacinamide concentration was not linear, as shown in the FID assay, but polynomial (Figure 8B).

Competition assays were also performed using a 505 bp *S. aureus nuc* fragment (30% GC) (Figure 8C,D). Although Hoechst fluorescence decreased with higher niacinamide concentration in a polynomial manner, the regression curve of this low-GC fragment was different from the *Bacillus* sp. fragment with 43% GC (Figure 8B). In the 43% GC fragment, the fluorescence decrease was higher in comparison to the 30% fragment and reached a plateau at 8% niacinamide; in addition, the standard deviations were lower in the 43% GC fragment, suggesting once again a preferable binding of niacinamide to GC over AT.

## 4. Discussion

Deciphering niacinamide’s binding mode to DNA is of outmost importance to our ability to fully understand its mechanism of action, as well as to harnessing its beneficial antimicrobial activity for various applications (e.g., a natural preservative in the cosmetics industry). In this study, we utilized well-recognized molecular assays such as melting curve analysis (Tm) and fluorescent intercalator displacement (FID) assays to investigate the mode of interaction of niacinamide with bacterial DNA.

Initially, melting curve analysis demonstrated that niacinamide interaction reduced the Tm of GC-rich bacterial dsDNA fragments in a dose-dependent and linear manner, indicating that niacinamide has a destabilizing effect on the double helix. Mono-intercalating compounds, such as ellipticine, adriamycin, and acridine orange (AO), all intercalate between two adjacent base pairs. With the help of intercalative rings, these molecules interact with the adjacent base pairs either in parallel or perpendicular ways, which unwind the DNA helix by an angle less than 36° (36° is the angle between two adjacent base pairs). Induction of local “unzipping” of the DNA helix by Ellipticine, adriamycin, and AO was shown to reduce the Tm by approximately 5 °C [17,18,19]. For comparison, in the presence of 6% niacinamide, the Tm of both *P. aeruginosa* 102 *ubi*B bp fragment and 198 bp *rpo*S fragment decreased by 5.25 °C and 4.5 °C, respectively, using the mono-intercalator SYBR Green as a reporter (Figure 2 and Figure 4). Therefore, niacinamide, as a small planar aromatic molecule, might also have a similar mode of interaction with dsDNA.

**Figure 9:**
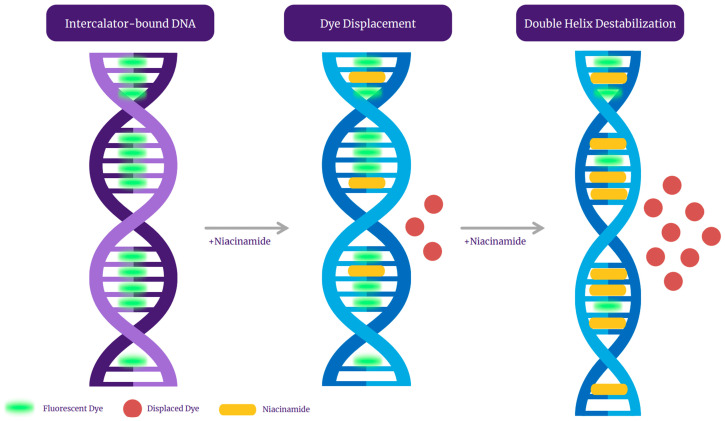
A graphical representation of niacinamide’s mode of interaction with intercalator dye-bound bacterial DNA.

Qualitative analysis of the melting peaks of bacterial dsDNA fragments in the presence of niacinamide also demonstrated a dose-dependent decrease in the height of the melt peak in the presence on niacinamide, which is consistent with partial denaturation of the dsDNA fragments by niacinamide, as well as dislocation of the fluorescent intercalating dye. Taken together, these observations, combined with the putative intercalative properties of niacinamide, has led us to propose “disruptive intercalation” and dye displacement as the mode of interaction for niacinamide with bacterial dsDNA (Figure 9). Such a putative mode of interaction is in agreement with previously published data, demonstrating that niacinamide binding to *Bacillus* sp. *gyr*B fragment resulted in band migration as well as a decrease in band intensity at a niacinamide concentration equal or superior to 30% [1]. Equally, Maikoo et al. previously concluded, based on a dose-dependent reduction in intensity of ethidium bromide (EtBr)-stained DNA, that a few ruthenium compounds were able to displace the DNA intercalator, EtBr [16].

The formation of locally destabilized DNA portions could interfere with protein/DNA recognition and potentially affect several crucial bacterial cellular processes, such as DNA repair, replication, and transcription, leading to cell death [17]. Thus, the newly observed mode of interaction of niacinamide with bacterial DNA can also explain its activity as an antimicrobial [1]. Out of the bacteria assessed, *P. aeruginosa* was found to be the most sensitive to niacinamide with a minimal inhibitory concentration (MIC_100_) of 15,000 ppm (1.5%), whereas *S. aureus* MIC_100_ was 25,000 ppm (2.5%) [1]. *P. aeruginosa* genome’s GC content is 65–67% [20], whereases the GC content of *S. aureus* typically ranges between 32.7% and 32.9% [21]. The substantial difference in niacinamide MIC and the genome content of these two bacteria encouraged us to investigate whether the ability of niacinamide to interact with bacterial DNA is GC content-dependent. As hypothesized, melting curve analysis, using *S. aureus* low-GC fragments demonstrated an overall same trend of decrease in Tm in the presence of niacinamide as with the high-GC fragments, but with a weaker correlation with niacinamide concentration, as evidenced by the difference in R^2^ values (0.73–0.86 for low-GC fragments, and >0.95 for high-GC fragments) (Figure 5 and Figure 6 vs. Figure 2, Figure 3 and Figure 4), suggesting a preference of binding of niacinamide to high-GC fragments. Indeed, intercalation preferentially occurs at GC-rich sequences (specifically CpG sites), as these sequences are unstacked easily [22]. Finally, niacinamide’s preferable intercalation to high-GC DNA can explain the previously observed difference in its antimicrobial efficacy against various bacteria.

The combined sequence composition and length determine the binding affinity, kinetics, and thermodynamics of intercalation [14,18,22]. The length of a DNA fragment influences the local sequence context available for intercalation, potentially impacting the binding affinity and the specificity. Longer sequences might provide additional or more varied binding sites, influencing the overall intercalation outcome. A study of bis-naphthalimide intercalators previously showed that the dynamics of intercalated rings, such as their rotational motions, depend on the length of the DNA duplex. Longer sequence length influences the flexibility and dynamic behavior of DNA during intercalation, affecting how intercalators bind and move within the DNA [23]. When comparing the slopes of the Tm regression plots of *P. aeruginosa* 102 bp *ubi*B and the 198 *rpo*S fragment (Figure 2D and Figure 4D, SYBR Safe), it is evident that the slope of the smaller fragment was ~2-fold-higher compared to the longer fragment (−4.2 vs. −2.1, respectively). The observed ratio is to be expected under a putative niacinamide intercalation model, as the smaller fragment contains approximately half of the potential niacinamide intercalation sites (i.e., base pairs) compared to the longer sequence. Accordingly, this observation further supports intercalation as niacinamide’s mode of interaction with bacterial DNA.

When analyzing the melting curves of *S. aureus* low-GC fragments, the *fem*B fragment demonstrated a higher correlation between Tm and the niacinamide concentration, compared to the *nuc* fragment (R^2^ = 0.86 vs. 0.73, respectively), despite the similarity of their GC contents (31% and 34%, respectively) (Figure 5 and Figure 6). The sequence analysis demonstrated that the *nuc* fragment has 10 purine stretches/100 bp, whereas the *fem*B fragment had six purine stretches/100 bp (Table 1). Purine bases (adenine and guanine) have aromatic rings, contributing to strong stacking interactions and influence on DNA folding and stability. Purine stretches can therefore affect the intercalation by changing the DNA conformation, base stacking, and local geometry, which in turn modulate how intercalators bind and distort the DNA duplex [23]. The difference in the number of purine stretches can result in the different intercalation kinetics of niacinamide to these fragments.

Intercalator displacement assays of known intercalators (e.g., methylene blue) by small, non-covalent, DNA-binding molecules, including the known intercalator Mitoxantrone [24], were previously applied to further support intercalation as the mode of interaction [16]. Similarly, we performed FID assays, using SYBR Safe stain, on both *Bacillus* sp. and *S. aureus* DNA fragments (42% and 30% GC, respectively) and demonstrated a dose-dependent decrease in fluorescence signal in the presence of niacinamide. The intercalator displacement rate was strongly correlated with the niacinamide concentration (Figure 7), further indicating the niacinamide’s ability to intercalate into the bacterial DNA fragment. Opposed to the FID assay data, results of low-GC fragment melting curve analysis in the presence of niacinamide did not demonstrate a linear response to niacinamide (Figure 5 and Figure 6), suggesting a preferable binding of niacinamide with GC-rich fragments. This apparent discrepancy in the results of the FID assay and melting curve analysis can be explained by the different analytical characteristics of these two methodologies. The FID assay is performed at RT, whereas the melting curve analysis is performed at 60–95 °C. Moreover, the FID is a kinetic assay measuring fluorescence over time, whereas the Tm is a single point, at which the DNA undergoes denaturation. Thus, it is possible that the melting curve analysis is a more sensitive method for demonstrating a preferable binding.

Under the intercalation working hypothesis, we initially expected niacinamide not to compete with Hoechst minor groove-binding dye, due to their discrete binding sites. Yet our data demonstrated a dose-dependent decrease in the fluorescence signal in the presence of niacinamide (Figure 8). This result might be attributed to structural changes in the double helix, following niacinamide intercalation. As mentioned before, intercalation can result in unwinding and lengthening of the double helix, consequently altering the conformation and size of the minor groove, thus hindering the binding of Hoechst dye to the DNA. Alternatively, similarly to other molecules, niacinamide can have a dual mode of interaction with the DNA. For example, Berenil, an anti-trypanosomal drug, is a di-cationic bis-benzamidine that has been categorized as a minor groove-binding ligand in AT-rich regions of the DNA. Studies have suggested that Berenil can bind to various DNA double strands exhibiting both intercalative and minor groove binding mode, depending upon the concentration [25]. Linear compounds might bind strongly in the minor groove if they can capture a terminal, interfacial water molecule that can complete the curvature of the compound. Such terminal water molecules can rapidly exchange with bulk water and form H-bonds between the niacinamide carboxamide group and the DNA, thus accounting for their minor entropy cost and significant binding enthalpy [26]. Nonetheless, the entire data presented here adamantly supports “disruptive intercalation” as the key mode of interaction behind niacinamide antimicrobial function (Figure 9).

## 5. Conclusions

We conclude based on melting curve analysis and fluorescent-based competition assays that niacinamide can intercalate into bacterial DNA, with a preference to GC content. Niacinamide intercalation results in a localized double helix destabilization, as well as the formation of denatured DNA regions. Consequently, all this correlating data strongly suggest that a “destabilizing intercalation” is the mode of binding behind the previously observed niacinamide antimicrobial activity.

## Figures and Tables

**Table 1:** Oligonucleotides and DNA fragments used in this study.

Target	Oligo Name	Oligo Sequence (5′-3′)	Amplicon Size (bp)	Amplicon GC Content (%)	Number of Purine Stretches *	Number of Purine Stretches/100 bp
*P. aeruginosa ubi*B	PAubiBF	AGGTCGCCCAACTGCATATC	102	62	7	6.9
	PAubiBR	TCTCGAAGATCG GTTCGCAG				
*P. aeruginosa rpoS*	rpoSF	CTCCCCGGGCAACCTCCAAAAG	198	59	10	5.1
	rpoSR	CGATCATCCGCTTCCGACCAG				
*S. aureus nuc*	nucSA-F	AAACAGCATCCTAAAAAAGGTGTA	180	34	18	10
	nucSA-R	AAGCTTCGTTTACCATTTTTCCATC				
*Bacillus safensis. gyr*B	gyrBSF_F1	TGCATTATCTACTACCTTAGACGTGAC	220	42	12	5.5
	gyrBSF_R2	TAAGAAAGCTAACTCACGAACACG				
*S. aureus nuc*	nucSA-F	AAACAGCATCCTAAAAAAGGTGTA	505	30	41	8.1
	nucSA-R2	CACATTGAACTATATAGTAACATTCC				
*Bacillus safensis. gyr*B	gyrBSF_F2	CATTATCAGCAGTTCAAACGCG	870	43	63	7.2
	gyrBSF_R4	TATTGTAGAGGGAGATTCTGCG				
*S. aureus fem*B	SAfemB-F	CGAATCGTGGTCCAGTAATGG	231	31	14	6.0
	SAfemB-R	CAGTTGTAAAGCCATGATGCTC				

* A purine stretch is defined as a string of at least 3 purines (A, G) framed on both sides by at least one pyrimidine (C, T).

## Data Availability

The original contributions presented in this study are included in the article/Supplementary Materials. Further inquiries can be directed to the corresponding authors.

## Supplementary Materials

The following supporting information can be downloaded at: https://www.mdpi.com/article/10.3390/microorganisms13071636/s1. Figure S1: Dye and niacinamide calibration for melting curve analysis; Figure S2: Dye and niacinamide calibration for competition assays; Figure S3: Sequence analysis for GC stretches.

## Abbreviations

The following abbreviations are used in this manuscript:

FID	Fluorescent intercalator displacement
AMP	Antimicrobial peptide
EtBr	Ethidium bromide
AO	Acridine orange
TSA	Tryptic soy agar
PCR	Polymerase chain reaction
bp	Base pairs
dsDNA	Double-strand DNA
TE	Tris-EDTA
Tm	Melting temperature
SD	Standard deviation
RT	Room temperature
MIC	Minimal inhibitory concentration
