# Supplementary material for: Disruptive DNA Intercalation Is the Mode of Interaction Behind Niacinamide Antimicrobial Activity
Source: Microorganisms. 2025 Jul 10;13(7):1636. doi: 10.3390/microorganisms13071636 (PMC12298274; doi:10.3390/microorganisms13071636)
Supplement: Supplementary file 1 [file microorganisms-13-01636-s001.zip › Supplementary Figure S1.pdf]

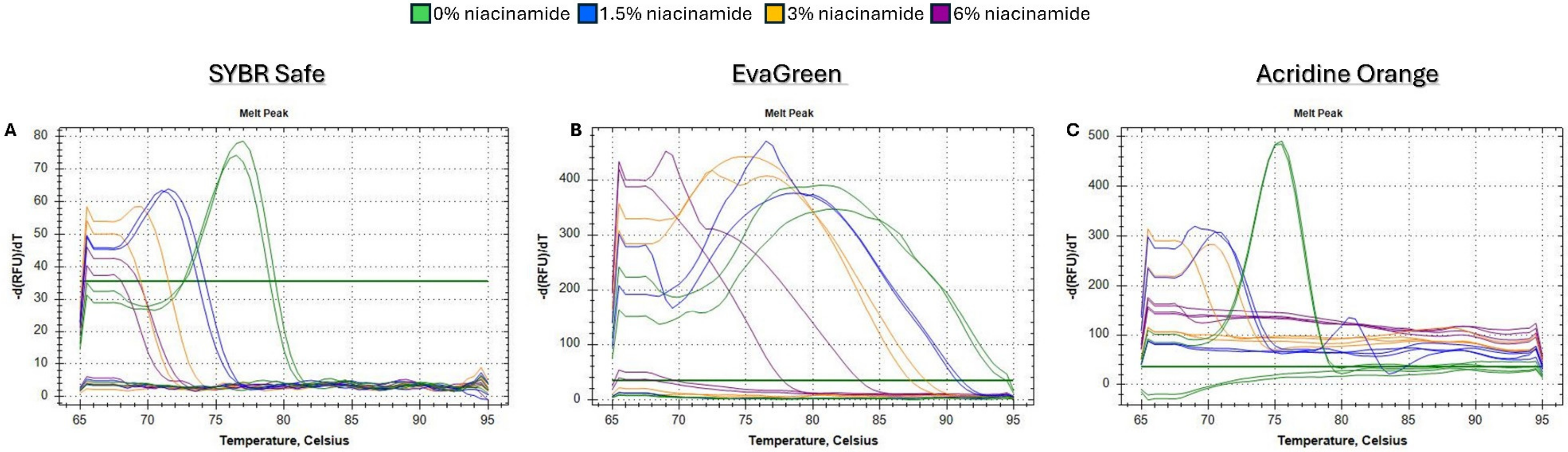

	Tm (°C)	
Niacinamide	SS	SS+DNA
0	ND	76.75
1.5%	ND	71.25
3.0%	ND	68.50
6.0%	ND	67.50

	Tm (°C)	
Niacinamide	EG	EG+DNA
0	ND	81.00
1.5%	ND	77.5
3.0%	ND	74.50
6.0%	ND	68.25

	Tm (°C)	
Niacinamide	AO	AO+DNA
0	ND	75.50
1.5%	ND	69.75
3.0%	ND	68.75
6.0%	ND	ND

Figure S1. Dye and niacinamide calibration for melting curve analysis. Melting curve analysis using intercalating fluorescent dyes SYBR™ safe (A), acridine orange (B), and EvaGreen® (C) as reporters, in the presence of niacinamide with or without 50 ng of *P. aeruginosa* 102 bp fragment (62 %GC). Shown are the melting peaks (upper panels) and melting temperature (lower panels) of a representative experiment performed in duplicate wells. ND-not detected.
