# Supplementary material for: Disruptive DNA Intercalation Is the Mode of Interaction Behind Niacinamide Antimicrobial Activity
Source: Microorganisms. 2025 Jul 10;13(7):1636. doi: 10.3390/microorganisms13071636 (PMC12298274; doi:10.3390/microorganisms13071636)
Supplement: Supplementary file 1 [file microorganisms-13-01636-s001.zip › Supplementary Figure S2.pdf]

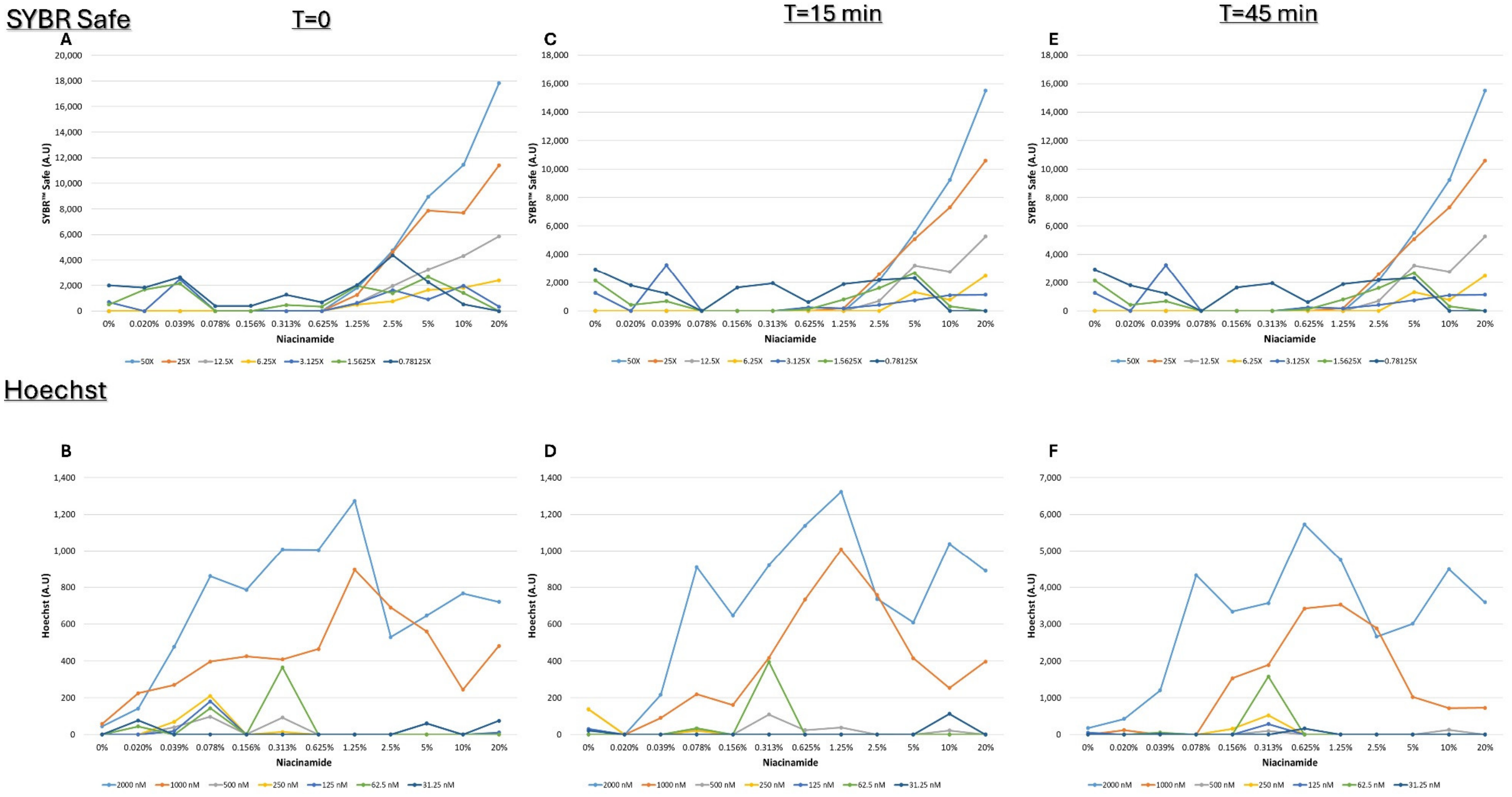

Figure S2. Dye and niacinamide calibration for competition assays. Niacinamide background interaction with DNA intercalating dye SYBR™ safe (A,C,E), and the minor-groove binding dye Hoechst (B,D,F), without DNA was measured at the indicated time points.
