# Supplementary material for: Disruptive DNA Intercalation Is the Mode of Interaction Behind Niacinamide Antimicrobial Activity
Source: Microorganisms. 2025 Jul 10;13(7):1636. doi: 10.3390/microorganisms13071636 (PMC12298274; doi:10.3390/microorganisms13071636)
Supplement: Supplementary file 1 [file microorganisms-13-01636-s001.zip › Supplementary Figure S3.pdf]

Supplementary Figure S3: Sequence analysis for GC stretches *

1. *Pseudomonas aeruginosa* (accession number CP081477.2)

***>Ps_ubi*B_102bp_fragment (%GC=62)**

AGGTCGCCCAACTGCATATCGACTCCGGCTGGGTACCGGCGGAAACCAAGGTCAACGACTTCGAGGCGGCGATCC
GCACCGTCTGCGAACCGATCTTCGAGA

***>Ps_rpoS_198*bp_fragment (59% GC)**

CTCCCCGGGCAACTCCAAAAGCCACCACTTCCTTCTCTTCCAAACAACACAAGCACATCGACTACACGCGCGCGTTG
GACGCAACGCAGCTGTATCTCAACGAAATCGGTTTCTCGCCCCTGTTGACGCCCGAAGAGGAAGTCCACTTCGCTC
GTCTGGCGCAGAAGGGCGATCCCGCTGGTCGGAAGCGGATGATCG

2. *Staphylococcus aureus* (accession number CP158284.1)

nuc fragments

>set1:nucSA-F-nucSA-R(180 bp)%GC=34%

AAACAAAGCATCCTAAAAAAGGTGTAGAGAAATATGGTCCTGAAGCAAGTGCATTTACGAAAAAAATGGTAGAA
AATGCAAAGAAAATTGAAGTCGAGTTTGACAAAGGTCAAAGAACTGATAAATATGGACGTGGCTTAGCGTATATT
TATGCTGATGGAAAAATGGTAAACGAAGCTT

>set2:nucSA-F-nucSA-R2(505 bp)%GC=30%

AAACAAAGCATCCTAAAAAAGGTGTAGAGAAATATGGTCCTGAAGCAAGTGCATTTACGAAAAAAATGGTAGAA
AATGCAAAGAAAATTGAAGTCGAGTTTGACAAAGGTCAAAGAACTGATAAATATGGACGTGGCTTAGCGTATATT
TATGCTGATGGAAAAATGGTAAACGAAGCTTTAGTTCGTCAAGGCTTGGCTAAAGTTGCTTATGTTTATAAACCTA
ACAATACACATGAACAACTTTTAAGAAAAAGTGAAGCACAAGCAAAAAAAGAGAAATTAAATATTTGGAGCGAAG
ACAACGCTGATTCAGGTCAATAATGCTCATTGTAAAAGTGTCACTGCTGCTAGTGGCACTTTTATAATTTTTAGATCA
CGATATGATTTATTATCAATTCAGAATTAAAAAAGTAAATAGTATCAAAAGTAAGTGTATTTAATATTAGAAAATAA
AAATTTTAAATTTAGTATTAAAATGGAATGTTACTATATAGTTCAATGTG

> *femB_*231bp*_fragment* (%GC=32)

CGAATCGTGGTCCAGTAATGGATTTTTCAGATTTAGGATTAGTTGATTATTATTTAAAAGAGTTAGATAAATATTTA
CAGCAACATCAATGTTTATATGTTAAATTAGATCCGTATTGGTTATATCATCTATATGATAAAGATATCGTGCCATTT
GAAGGTCGCGAGAAAAATGATGCCTTAGTAAACTTGTTTAAATCACATGGTTACGAGCATCATGGCTTTACAACTG

3. *Bacillus sp*

***gyr*B fragments**

>set1:gyrBSF_F2-gyrBSF_R4(870 bp)%GC=43 (accession number AY167878.1)

CATTATCAGCAGTTCAAACGCGGCGTTCCAGTGGGAGATTTAGAGGTTATTGGTGAAACTGATGTAACCGGGACA
ACCACTCACTTTGTGCCAGATCCAGAAATTTTCACGGAAACCATTGAATTTGATTACGATACACTTGCTAACCGTGTT
CGTGAGTTAGCTTTCTTAACAAAAGGTGTAAACATCATCATAGAAGACTTGCGTGAAGGCAAAGAGCGGAGAAAT
GAATACTGCTACGAAGGCGGTATTAAGAGCTATGTAGAACATTTAAACCGCTCAAAAGAAGTCGTTCATGAAGAA
CCTGTGTACATCGAAGGTGAGAAAGACGGAATCACCGTTGAAGTAGCATTACAATATAACGATTCCTATACAAGCA
ATATCTATTCCTTCGCTAACAATATCAACACGTATGAAGGCGGAACACACGAAGCTGGTTTTAAAACCGGTCTAACG

CGTGTCATCAATGACTATGCTCGTAAAAATGGCGTATTCAAAGATGGGGATGCGAATTTAAGTGGTGAAGATGTG
CGAGAAGGCTTAACAGCCATTATCTCCATCAAACATCCAGACCCTCAATTCGAAGGACAAACGAAGACAAAGCTT
GGGAACTCAGAAGCGAGAACCATCACAGACTCCCTTTTCTCTGAAGCACTTGAGAAATTCTTGCTTGAAAATCCTG
ATTCTGCGAAAAAAATTGTGGAAAAAGGACTGATGGCAGCTCGTGCAAGAATGGCTGCCAAAAAGGCTCGTGAGC
TGACAAGACGTAAAAGTGCACTGGAAGTCTCCAGCTTACCTGGGAAACTGGCGGACTGTTCTTCTAAAGATCCTTC
CATCTCTGAACTCTATATTGTAGAGGGAGATTCTGCG

>set2:gyrBSF_F1-gyrBSF_R2(220 bp)%GC=43 (accession number CP152027.1)

TGCATTATCTACTACCTTAGACGTGACCGTATACCGTGACGGAAAAATTCATTATCAGCAGTTCAAACGCGGTGTTC
CAGTGGGAGATTTAGAGGTTATTGGTGAAACAGATGTAACCGGGACAACAACCCACTTTGTGCCAGATCCAGAAA
TTTTCACGGAAACCATTGAATTTGATTACGATACACTTGCTAACCGTGTTCGTGAGTTAGCTTTCTTA

* Purine stretches are highlighted by box borders. A purine stretch is defined as a string of at least 3 purines (A, G) framed on both sides by at least one pyrimidine (C, T).
